# Comparing scientific abstracts generated by ChatGPT to real abstracts with detectors and blinded human reviewers

**DOI:** 10.1038/s41746-023-00819-6

**Published:** 2023-04-26

**Authors:** Catherine A. Gao, Frederick M. Howard, Nikolay S. Markov, Emma C. Dyer, Siddhi Ramesh, Yuan Luo, Alexander T. Pearson

**Affiliations:** 1 Division of Pulmonary and Critical Care, Department of Medicine, Northwestern University Feinberg School of Medicine, Chicago, IL USA; 2 Section of Hematology/Oncology, Department of Medicine, University of Chicago, Chicago, IL USA; 3 Division of Health and Biomedical Informatics, Department of Preventive Medicine, Northwestern University Feinberg School of Medicine, Chicago, IL USA

**Keywords:** Publishing, Medical research, Computational biology and bioinformatics

## Abstract

Large language models such as ChatGPT can produce increasingly realistic text, with unknown information on the accuracy and integrity of using these models in scientific writing. We gathered fifth research abstracts from five high-impact factor medical journals and asked ChatGPT to generate research abstracts based on their titles and journals. Most generated abstracts were detected using an AI output detector, ‘GPT-2 Output Detector’, with % ‘fake’ scores (higher meaning more likely to be generated) of median [interquartile range] of 99.98% ‘fake’ [12.73%, 99.98%] compared with median 0.02% [IQR 0.02%, 0.09%] for the original abstracts. The AUROC of the AI output detector was 0.94. Generated abstracts scored lower than original abstracts when run through a plagiarism detector website and iThenticate (higher scores meaning more matching text found). When given a mixture of original and general abstracts, blinded human reviewers correctly identified 68% of generated abstracts as being generated by ChatGPT, but incorrectly identified 14% of original abstracts as being generated. Reviewers indicated that it was surprisingly difficult to differentiate between the two, though abstracts they suspected were generated were vaguer and more formulaic. ChatGPT writes believable scientific abstracts, though with completely generated data. Depending on publisher-specific guidelines, AI output detectors may serve as an editorial tool to help maintain scientific standards. The boundaries of ethical and acceptable use of large language models to help scientific writing are still being discussed, and different journals and conferences are adopting varying policies.

The release of OpenAI’s free tool ChatGPT^1^ on November 30, 2022 demonstrated the ability of artificial intelligence models to generate content, with articles quickly published on its possible uses and potential controversies^2–4^. Early adopters have shared their experiences on social media, with largely positive sentiments^5^. Articles are bemoaning the death of the traditional school essay assignment^4,6,7^, as ChatGPT has been shown to generate high-scoring papers^8^, correctly answer USMLE questions^9^, and even articulate critical thinking^10^. The ethical and acceptable boundaries of ChatGPT’s use in scientific writing remain unclear^11^, although some publishers are beginning to lay down policies^12–14^.

Large language models (LLM) are often complex neural network-based transformer models that can generate tone and content-defined text. These are trained on enormous amounts of data to predict the best next text element, which produces a product that reads naturally. ChatGPT is built on Generative Pre-trained Transformer-3 (GPT-3), which is one of the largest of these types of models, trained with 175 billion parameters^15^. These models generate coherent and fluent output, that can be difficult to distinguish from text written by humans^16,17^.

Artificial intelligence (AI) has numerous applications in medical technologies^18^, and the writing of biomedical research is no exception, with products such as the SciNote Manuscript Writer^19^ or Writefull^20^ that help with scientific writing. However, with the release of ChatGPT, this powerful LLM technology is now available to all users for free, and millions are engaging with the new technology. The user base is likely to continue to grow. Thus, there is an urgent need to determine if ChatGPT can write convincing medical research abstracts.

We gathered 50 abstracts from five high-impact journals as our control corpus of well-written abstracts. We asked ChatGPT to generate 50 scientific abstracts based on the titles and specific journals from this list (example subset in Supplementary Data 1). While all the output appeared superficially to be formatted as a scientific abstract, only 8 (16%) correctly used the headings particular to the specific journal in the prompt (e.g., *Nature Medicine’*s paragraph-style without headings, as opposed to specific headings such as ‘Design, Setting, and Participants’ for *JAMA*, see Supplementary Note 1 for examples). The patient cohort sizes were a similar order of magnitude between the original abstracts and the generated abstracts, with a Pearson correlation of the logarithmic cohort sizes of *r* = 0.76, *p* < 0.001 (Fig. 1).

**Fig. 1:**
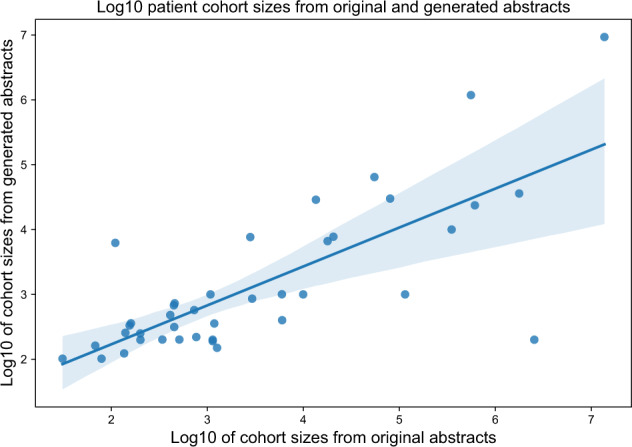
Generated abstracts have a similar patient cohort size as original abstracts. Cohort sizes from original abstracts (*x*-axis) and generated abstracts (*y*-axis) plotted on a logarithmic 10 scale.

The AI output detector we used, ‘GPT-2 Output Detector’^21,22^, found a high probability of AI-generated output (higher % ‘fake’ score indicating more likely to be AI-generated text) in the generated abstracts with median [IQR] of 99.98% [12.73%, 99.98%] compared with very low probability of AI-generated output in nearly all the original abstracts with median [IQR] of 0.02% [0.02%, 0.09%] (Fig. 2a). The AI output detector had an area under the receiver operating characteristics (AUROC) curve of 0.94 for detecting generated abstracts (Fig. 2b). At the optimal cutoff maximizing sensitivity and specificity, 1.46%, the AI output detector had a sensitivity of 86% and a specificity of 94% at differentiating original versus generated abstracts.

**Fig. 2:**
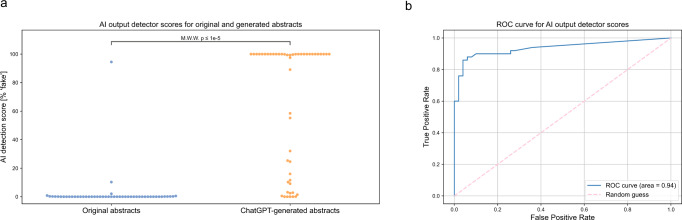
Many generated abstracts can be detected using an AI output detector. **a** AI detection scores as [% ‘fake’] per GPT-2 Output Detector for original abstracts and generated abstracts. Higher score indicates more likely to be generated by AI. **b** The AI output detector ROC curve for discriminating between original and generated abstracts, with AUROC of 0.94.

We ran both original and generated abstracts through a free plagiarism-detection website, Plagiarism Detector^20^, and a paid professional similarity checker, iThenticate^23^. For both platforms, a higher score indicates more matching text was found. Original abstracts scored higher on the Plagiarism Detector website with median ‘plagiarized’ score 62.5% [IQR 43.25%, 84.75%] compared with generated abstracts with median ‘plagiarized’ score of 0% [IQR 0, 0] (Fig. 3a). Original abstracts also scored higher on iThenticate with median similar index of 100 [IQR 100, 100] compared with generated abstracts that had with median similarity index of 27 [IQR 19, 40.75] (Fig. 3b).

**Fig. 3:**
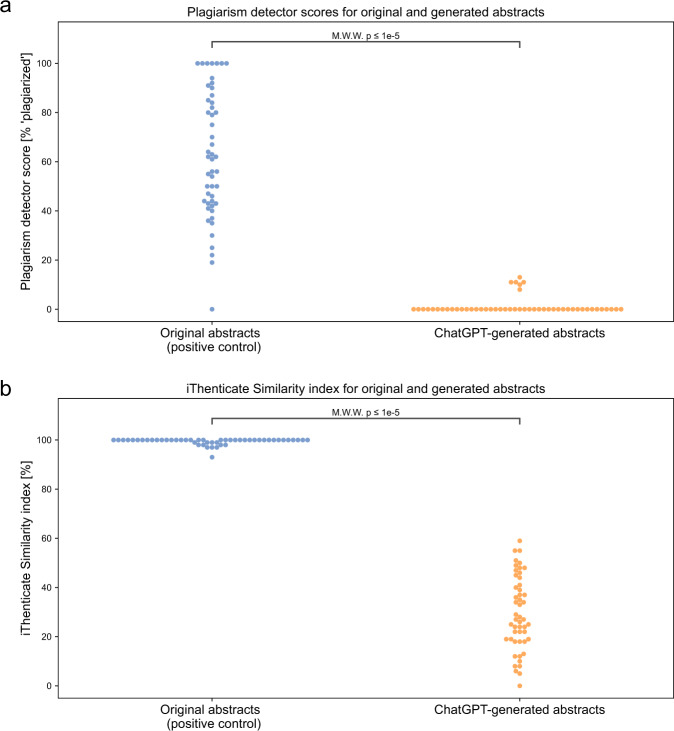
Generated abstracts score lower than original abstracts on plagiarism detectors. **a** Plagiarism scores from plagiarism detector website, with higher % ‘plagiarized’ score indicating more matching text was found. **b** iThenticate Similarity Index for original abstracts and generated abstracts [%], with higher value meaning more similar text was found.

Blinded human reviewers (FMH, NSM, ECD, SR) were given a mixture of real and generated abstracts and asked to provide a binary score of real or generated (Table 1). They were able to correctly identify 68% of generated abstracts as being generated, and correctly identified 86% of original articles as being original. They incorrectly identified 32% of generated abstracts as being real, and 14% of original abstracts as being generated. Our reviewers commented that abstracts they thought were generated by ChatGPT were superficial and vague, and sometimes focused on details of original abstracts such as inclusion of Clinical Trial Registration numbers and alternative spellings of words. The AI output detector scores were not statistically different (*p* = 0.45 by MWW) between the abstracts that reviewers correctly identified as generated and ones that they failed to identify as generated (Fig. 4).

**Table 1:** Human reviewer scoring for whether abstracts were real or generated, along with truth.

		Truth	
		Original	Generated
Reviewer guess	Original	43	16
	Generated	7	34

**Fig. 4:**
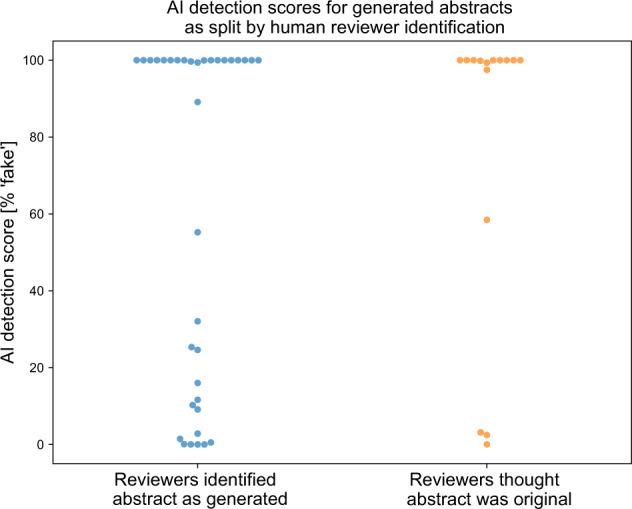
Reviewers use criteria different than the AI output detector for flagging abstracts as either generated or original. The AI detection scores for generated abstracts were not significantly different (*p* = 0.45) between abstracts that human reviewers identified as generated, and those that they failed to identify as generated.

In this study, we found that both humans and AI output detectors were able to identify a portion of abstracts generated by ChatGPT, but neither were perfect discriminators. Our reviewers even misclassified a portion of real abstracts as being generated, indicating they were highly skeptical when reviewing the abstracts. The generated abstracts contained fabricated numbers but were in a similar range as the real abstracts; ChatGPT knew from its training data that studies on hypertension should include a much larger patient cohort size than studies on rarer diseases such as monkeypox.

Limitations to our study include its small sample size and few reviewers. ChatGPT is also known to be sensitive to small changes in prompts; we did not exhaust different prompt options, nor did we deviate from our prescribed prompt. ChatGPT generates a different response even to the same prompt multiple times, and we only evaluated one of infinite possible outputs. We took only the first output given by ChatGPT, without additional refinement that could enhance its believability or improve its escape from detection. Thus, our study likely underestimates the ability of ChatGPT to generate scientific abstracts. The maximum input for the AI output detector we used is 510 tokens, thus some of the abstracts were not able to be fully evaluated due to their length. Our study ream reviewers knew that a subset of the abstracts they were viewing were generated by ChatGPT, but a reviewer outside this context may not be able to recognize them as written by a large language model. We only asked for a binary response from our reviewer team of original or generated and did not use a formal or more sophisticated rubric. Future studies could expand on our methodology to include other AI output detector models, other plagiarism detectors, more formalized review, as well as text from other fields outside of biomedical sciences.

We anticipate that this technology could be used in both an ethical and unethical way. Given its ability to generate abstracts with believable numbers, it could be used by organizations such as paper mills to entirely falsify research. On the other hand, the technology may be used in conjunction with a researcher’s own scientific knowledge as a tool to decrease the burden of writing and formatting. It could be used by scientists publishing in a language that is not their native language, to improve equity. However, AI models have been shown to be highly sensitive to biases in training data^24,25^, and further data is needed to determine the potential for bias perpetuated by ChatGPT—especially given the overt prejudices emerging from prior language generation models^26^.

We suggest clear disclosure when a manuscript is written with assistance from large language models such as ChatGPT. Though there is ongoing work to embed watermarks in AI-generated output, it is unknown when this will be standardized and robust against scrubbing efforts. Reassuringly, there are patterns that allow it to be detected by AI output detectors, although there has been exploration of techniques to fool AI output detectors^27^. Different journals and publishers are developing their own policies on whether large language models can contribute to the writing of papers, ranging from not allowing any AI-generated text^12^ to allowing its use as long as it is openly disclosed^13,14,28^. Though imperfect, AI output detectors may be one tool to include in the research editorial process, depending on the publisher or conference’s guidelines.

Abstract generation by large language models such as ChatGPT is a powerful tool to create readable scientific abstracts, though includes generated data. The generated abstracts do not always alarm plagiarism-detection models, as the text is generated anew, but can often be detected using AI detection models, and sometimes identified by a person. Generated text may help alleviate the burden of writing by providing an outline for a scientist to edit but requires careful review for factual accuracy. The optimal use and ethical boundaries of AI-generated writing remain to be determined as discussion within the scientific community evolves.

## Methods

### Abstract generation

We evaluated the abstracts generated by ChatGPT (Version Dec 15) for 50 scientific medical papers. We gathered titles and original abstracts from current and recent issues (published in late November and December of 2022) of five high-impact journals (*Nature Medicine, JAMA*, *NEJM*, *BMJ*, *Lancet*) and compared them with the original abstracts. The prompt fed to the model was ‘Please write a scientific abstract for the article [title] in the style of [journal] at [link]’. Note that the link is superfluous because ChatGPT cannot browse the internet. ChatGPT’s knowledge cutoff date is September 2021. We ran each prompt in a new session.

### Abstract evaluation

We evaluated the ChatGPT-generated abstracts for plagiarism detection using a free web-crawling plagiarism-detection tool ‘Plagiarism Detector’^29^, which gives a ‘plagiarized’ score from 0–100%, with a higher score indicating that more plagiarism was detected; these analyses were run in December. We also evaluated the abstracts using a paid similarity checker program, iThenticate^23^, which outputs a 0–100% ‘similarity index’, with a higher score indicating more redundant with existing text. We also evaluated abstracts with an AI output detector using the ‘GPT-2 Output Detector’^21,22^, a RoBERTa-based sequence classifier, which gives abstracts a score ranging from 0.02 to 99.98% ‘fake’, with a higher score indicating the text was more likely to be generated by an AI algorithm.

We evaluated whether blinded human reviewers (study team members FMH, NSM, ECD, SR, members of our biomedical sciences laboratories used to reading scientific abstracts) could identify ChatGPT-generated abstracts. For every pair of reviewers, we used randomization via an electronic coin flip to decide whether an original or generated abstract would be provided for the first reviewer, with the opposite being given to the second reviewer. Each reviewer was given 25 abstracts to review, informed that there was a mixture of original and generated abstracts, asked to give a binary score of whether they thought the abstract was original or generated and invited to make free-text observations while reviewing. Reviewers were not shown any data or analysis until after their scoring of abstracts was completed.

We gave a binary yes/no score of whether the format of the generated abstract adhered to the journal’s requirements by comparing it to the original article’s headings and structure. We also compared the reported patient cohort sizes between the original and generated abstracts with a Pearson correlation of the logarithmic cohort sizes.

### Statistics and visualization

Graphics and statistics were done in Python version 3.9 with *seaborn* version 0.11.2^30^, *matplotlib* version 3.5.1^31^, *sklearn* version 1.0.2^32^, *scipy* version 1.7.3^33^, and *statsannotations* version 0.4.4^34^. Group statistics are reported using median [interquartile range] and were compared using two-sided Mann Whitney Wilcoxon (MWW) tests, with *p* < 0.05 being the cutoff for statistical significance. Proportions were compared with Fisher’s Exact tests. Correlation between the cohort sizes was done with Pearson’s correlation.

### Reporting summary

Further information on research design is available in the Nature Research Reporting Summary linked to this article.

## Supplementary information

Supplementary Information

REPORTING SUMMARY

## Data Availability

The data used in the manuscript are available upon reasonable request to the corresponding author.

## Code availability

The code used in the manuscript are available upon reasonable request to the corresponding author.
