# Supplementary material for: Comparing scientific abstracts generated by ChatGPT to real abstracts with detectors and blinded human reviewers
Source: NPJ Digit Med. 2023 Apr 26;6:75. doi: 10.1038/s41746-023-00819-6 (PMC10133283; doi:10.1038/s41746-023-00819-6)
Supplement: Supplementary file 1 — Supplementary Information [file 41746_2023_819_MOESM1_ESM.pdf]

**Supplemental Materials.**

**Supplementary Data 1.** An example subset of the generated abstracts.

"Atezolizumab plus anthracycline-based chemotherapy in metastatic triple-negative breast cancer: the randomized, double-blind phase 2b ALICE trial"[1] in the style of Nature Medicine

Triple-negative breast cancer (TNBC) is a subtype of breast cancer that is aggressive and difficult to treat. Atezolizumab is a monoclonal antibody that targets the protein PD-L1, which is expressed on some cancer cells and can help them evade the immune system. In this double-blind, phase 2b clinical trial, called ALICE, we evaluated the safety and efficacy of adding atezolizumab to an anthracycline-based chemotherapy regimen in patients with metastatic TNBC. A total of 162 patients were randomized to receive either atezolizumab plus chemotherapy or placebo plus chemotherapy. The primary endpoint was progression-free survival (PFS), which was significantly longer in the atezolizumab group compared to the placebo group (median PFS of 6.3 months vs 3.7 months, respectively; hazard ratio 0.59, 95% confidence interval 0.43-0.81, p=0.0007). Overall survival and objective response rate were also higher in the atezolizumab group, although the differences did not reach statistical significance. Adverse events were similar between the two groups, with the most common being neutropenia, anemia, and nausea. In conclusion, the addition of atezolizumab to anthracycline-based chemotherapy significantly improves PFS in patients with metastatic TNBC, with a manageable safety profile. These findings support the further investigation of atezolizumab in this patient population.

“Association of COVID-19 Vaccinations With Intensive Care Unit Admissions and Outcome of Critically Ill Patients With COVID-19 Pneumonia in Lombardy, Italy”[2] in the style of JAMA

Background: COVID-19 has had a significant impact on healthcare systems worldwide, leading to high rates of intensive care unit (ICU) admissions and mortality. Vaccines have been developed and deployed as a means to combat the pandemic, but their effect on critically ill patients with COVID-19 pneumonia remains unclear.

Objective: To determine the association of COVID-19 vaccinations with ICU admissions and outcomes in critically ill patients with COVID-19 pneumonia in Lombardy, Italy.

Design: Retrospective cohort study.

Setting: ICUs in Lombardy, Italy.

Participants: A total of 314 critically ill patients with COVID-19 pneumonia admitted to ICUs in Lombardy between January 1 and June 30, 2021.

Exposure: COVID-19 vaccination status.

Main Outcomes and Measures: The primary outcome was ICU admission, and secondary outcomes included ICU mortality and length of ICU stay.

Results: Of the 314 critically ill patients with COVID-19 pneumonia, 149 (47.5%) were vaccinated. The vaccinated group had a significantly lower rate of ICU admission compared with the unvaccinated group (47.0% vs 63.2%; difference, -16.2% [95% CI, -26.6% to -5.9%]; P = .002). In addition, vaccinated patients had a lower ICU mortality rate compared with unvaccinated patients (14.8% vs 25.0%; difference, -10.2% [95% CI, -20.8% to 0.3%]; P = .05). The length of ICU stay did not differ significantly between the vaccinated and unvaccinated groups (median, 8 days [interquartile range, 5-13 days] vs 9 days [interquartile range, 5-14 days]; P = .50).

Conclusions and Relevance: In this cohort of critically ill patients with COVID-19 pneumonia, vaccination was significantly associated with lower rates of ICU admission and mortality. These findings suggest that COVID-19 vaccines may provide a potentially important benefit for critically ill patients with COVID-19 pneumonia.

“Once-Weekly Semaglutide in Adolescents with Obesity”[3] in the style of NEJM

Objective: To evaluate the efficacy and safety of once-weekly semaglutide, a glucagon-like peptide-1 receptor agonist, in adolescents with obesity.

Methods: This was a randomized, double-blind, placebo-controlled, parallel-group trial conducted at 29 clinical centers in the United States and Canada. A total of 199 adolescents aged 12 to <18 years with a body mass index ≥35 kg/m2 or ≥30 kg/m2 with comorbidities were randomized to receive once-weekly subcutaneous injections of semaglutide (1.0 or 0.5 mg) or placebo for 68 weeks. The primary outcome was the change in body weight from baseline to week 68.

Results: At week 68, the mean change in body weight was -11.9 kg in the semaglutide 1.0-mg group, -9.5 kg in the semaglutide 0.5-mg group, and -2.5 kg in the placebo group (P < 0.001 for both semaglutide groups vs. placebo). The proportion of participants who lost ≥5% and ≥10% of their body weight was greater in the semaglutide groups than in the placebo group (P < 0.001 for both comparisons). Semaglutide was associated with statistically significant improvements in blood pressure, glycemic control, and lipid profiles. The incidence of adverse events was similar in the semaglutide and placebo groups, with no significant differences in the incidence of serious adverse events or serious treatment-emergent adverse events.

Conclusions: In this randomized trial, once-weekly semaglutide was effective and well tolerated in adolescents with obesity, leading to clinically meaningful and statistically significant weight loss and improvements in cardiometabolic risk factors.

Trial Registration: ClinicalTrials.gov, NCT03702497.

“Racial disparities in recommendations for surgical resection of primary brain tumours: a registry-based cohort analysis”[4] in the style of The Lancet

Introduction: Despite progress in the treatment of primary brain tumours, racial disparities in patient outcomes persist. Previous studies have suggested that these disparities may be related to differences in treatment recommendations and access to care. However, the extent to which racial disparities exist in the recommendation of surgical resection for primary brain tumours is unclear.

Methods: We conducted a registry-based cohort analysis using data from the National Cancer Database to examine racial disparities in recommendations for surgical resection of primary brain tumours in the United States. We included patients diagnosed with a primary brain tumour between 2010 and 2014, and excluded those with metastatic disease or missing race/ethnicity data. We compared the proportion of white and non-white patients who received a recommendation for surgical resection, adjusting for patient, tumour, and hospital characteristics.

Results: Among the 23,632 patients included in the analysis, 63.4% were white and 36.6% were non-white. After adjusting for patient, tumour, and hospital characteristics, non-white patients were significantly less likely to receive a recommendation for surgical resection than white patients (odds ratio 0.87, 95% CI 0.81-0.93).

Conclusion: Our study found significant racial disparities in the recommendation of surgical resection for primary brain tumours in the United States. Further research is needed to identify the causes of these disparities and to develop interventions to reduce them.

“Efficacy of awake prone positioning in patients with covid-19 related hypoxemic respiratory failure: systematic review and meta-analysis of randomized trials”[5] in the style of The BMJ

Objective: To evaluate the efficacy of awake prone positioning (APP) in patients with COVID-19 related hypoxemic respiratory failure.

Design: Systematic review and meta-analysis of randomized controlled trials (RCTs).

Data sources: A comprehensive search was conducted in multiple databases including PubMed, Embase, and the Cochrane Library, up to June 2021.

Eligibility criteria: RCTs that compared APP with standard care in patients with COVID-19 related hypoxemic respiratory failure were included.

Data extraction and synthesis: Two reviewers independently extracted data and assessed the risk of bias. The primary outcome was oxygenation index (OI), defined as the ratio of partial pressure of arterial oxygen (PaO2) to fraction of inspired oxygen (FiO2). The secondary outcomes were 28-day mortality, duration of mechanical ventilation, and length of hospital stay. The meta-analysis was performed using a fixed-effect model.

Results: Six RCTs with a total of 859 patients were included in the final analysis. The meta-analysis showed that APP significantly improved OI compared to standard care (mean difference [MD] -10.65, 95% confidence interval [CI] -15.29 to -5.99, $p < 0.001$). There was also a trend towards a lower 28-day mortality in the APP group (relative risk [RR] 0.63, 95% CI 0.40 to 0.98, $p = 0.04$). There were no significant differences in the duration of mechanical ventilation (MD -1.40, 95% CI -3.90 to 1.11, $p = 0.27$) or length of hospital stay (MD -2.43, 95% CI -6.69 to 1.82, $p = 0.28$) between the two groups.

Conclusion: This systematic review and meta-analysis suggests that APP may be effective in improving oxygenation and reducing 28-day mortality in patients with COVID-19 related hypoxemic respiratory failure. Further high-quality RCTs are needed to confirm these findings.

**Supplementary Note 1**. Formatting header specifications noted for journals *Nature Medicine, JAMA, NEJM, BMJ,* and *The Lancet.*

*Nature Medicine* - headerless paragraph abstract

*JAMA*
Importance
Objective
Design, Setting, and Participants
Interventions
Main Outcomes and Measures
Results
Conclusions and Relevance

*The NEJM*
Background
Methods
Results
Conclusions

*The BMJ* (specific headers variable depending on type of study)
Objective
Design
Data sources
Eligibility criteria
Setting
Participants
Data extraction and synthesis
Main outcome measures
Results
Conclusions

*The Lancet*
Background
Methods
Findings
Interpretation

Works Cited


1. Røssevold, A. H. *et al.* Atezolizumab plus anthracycline-based chemotherapy in metastatic triple-negative breast cancer: the randomized, double-blind phase 2b ALICE trial. *Nat. Med.* 1–11 (2022).
2. Grasselli, G. *et al.* Association of COVID-19 vaccinations with intensive care unit admissions and outcome of critically ill patients with COVID-19 pneumonia in Lombardy, Italy. *JAMA Netw. Open* **5**, e2238871 (2022).
3. Weghuber, D. *et al.* Once-weekly semaglutide in adolescents with obesity. *N. Engl. J. Med.* **387**, 2245–2257 (2022).
4. Butterfield, J. T. *et al.* Racial disparities in recommendations for surgical resection of primary brain tumours: a registry-based cohort analysis. *Lancet* **400**, 2063–2073 (2022).
5. Weatherald, J. *et al.* Efficacy of awake prone positioning in patients with covid-19 related hypoxemic respiratory failure: systematic review and meta-analysis of randomized trials. *BMJ* **379**, e071966 (2022).
